# Exogenous 24-Epibrassinolide Enhances NaHCO_3_ Tolerance in Cucumber Seedlings Associated with Changes in Carbohydrate Metabolism, Glycolysis, and the Tricarboxylic Acid Cycle

**DOI:** 10.3390/plants15213263

**Published:** 2026-10-22

**Authors:** Wenjing Nie, Qinghua Shi, Yinyu Gu, Weijun Yin, Ruxia Li, Zhihua Qiao, Haiman Ge, Min Liu, Junlin Li, Dongyang Li, Xiangyu Wang, Chi Zhang

**Affiliations:** 1 Yantai Engineering Research Center for Plant Stem Cell Targeted Breeding, Shandong Engineering Research Center of Functional Crop Germplasm Innovation and Cultivation Utilization, Shandong Institute of Sericulture, Yantai 265503, China; cottage1990@163.com (W.N.);; 2 College of Horticulture Science and Engineering, Shandong Agricultural University, No. 61 Daizong Street, Tai’an 271018, China; 3 National Center of Technology Innovation for Comprehensive Utilization of Saline-Alkali Land, Dongying 265503, China

**Keywords:** cucumber, 24-epibrassinolide, NaHCO_3_ stress, carbohydrate metabolism, glycolysis, tricarboxylic acid cycle

## Abstract

This study evaluated the role of exogenous 24-epibrassinolide (EBR) in mitigating NaHCO_3_ stress in cucumber (*Cucumis sativus* L.) seedlings, with particular attention to carbohydrate metabolism, glycolysis, and the tricarboxylic acid (TCA) cycle. Seedlings were exposed to 30 mM NaHCO_3_ with or without 0.2 μM EBR, and physiological and metabolic responses were evaluated during a 15-d treatment period, with gene expression analyzed in roots at 24 h. EBR treatment substantially mitigated the adverse effects of NaHCO_3_, as evidenced by improved cucumber growth, reduced inhibition of photosynthetic rate (Pn) and Fv/Fm, and increased biomass accumulation. NaHCO_3_ stress caused excessive starch accumulation in leaves while reducing starch levels in roots, indicating marked alterations in carbohydrate accumulation in the two tissues. EBR partially mitigated these changes by reducing starch accumulation in leaves while increasing starch content in roots. In addition, EBR also altered the activities of sucrose-metabolizing enzymes. NaHCO_3_ stress inhibited key glycolytic enzymes, including phosphofructokinase (PFK) and pyruvate kinase (PK), and suppressed the activities of isocitrate dehydrogenase (IDH) and succinate dehydrogenase (SDH) during prolonged stress. These inhibitory effects were partially alleviated by EBR, which also altered organic acid accumulation under NaHCO_3_ stress. Transcript analysis further showed that EBR altered the expression patterns of genes associated with glycolysis and the TCA cycle, including *HK*, *PFK*, *PK*, *IDH*, *SDH*, *MDH*, and *FUM*. Taken together, these findings indicate that EBR improves cucumber tolerance to NaHCO_3_ stress in association with adjustments in carbohydrate, glycolytic, and TCA-cycle metabolism, together with improved biomass accumulation.

## 1. Introduction

Saline–alkali stress severely restricts cucumber growth and productivity and represents one of the major abiotic constraints on agricultural production worldwide [1,2]. Compared with neutral salt stress, saline–alkali stress causes not only ionic toxicity and osmotic stress but also high-pH injury [3,4], which exerts more severe adverse effects on plant growth and development. Excessive accumulation of Na^+^ and HCO_3_^−^/CO_3_^2−^ disrupts nutrient uptake [5], impairs photosynthesis, disturbs metabolic homeostasis, and ultimately reduces crop productivity. Cucumber (*Cucumis sativus* L.) is an economically important vegetable crop that is highly sensitive to saline–alkali conditions [6]. Exposure to NaHCO_3_ stress markedly inhibits seedling growth and reduces biomass accumulation. It also causes leaf chlorosis and wilting and decreases photosynthetic parameters such as net photosynthetic rate (Pn) and maximal photochemical efficiency of PSII (Fv/Fm) [7]. It also disrupts root morphology and membrane integrity, leading to significant declines in overall plant performance. These effects highlight the urgent need to elucidate the physiological and metabolic mechanisms underlying cucumber adaptation to saline–alkali stress and to develop effective tolerance-enhancing strategies for sustaining production in affected areas [2,5].

Photosynthesis is the primary source of carbon assimilation in plants and provides carbohydrates required for growth, respiration, and stress responses [8,9]. Under saline–alkali stress, photosynthetic carbon assimilation is frequently inhibited due to stomatal limitation, chlorophyll degradation, and reduced PSII efficiency [1,10]. These changes may affect carbohydrate synthesis, accumulation, and utilization and potentially alter source–sink relationships between leaves and roots [11]. Sucrose, glucose, fructose, and starch are key components of plant carbon metabolism [11]. Their accumulation is regulated by carbohydrate-metabolizing enzymes, including invertases (INV), sucrose synthase (SS), and sucrose-phosphate synthase (SPS) [11]. Saline–alkali stress can induce marked changes in starch contents in leaves and roots, reflecting stress-associated alterations in carbohydrate metabolism [8,9]. Soluble sugars serve not only as metabolic substrates and osmotic regulators but also as signaling molecules in stress adaptation pathways [12]. Furthermore, glycolysis together with the tricarboxylic acid (TCA) cycle constitutes a major downstream component of carbon metabolism [13]. Glycolysis generates pyruvate, which can be converted to acetyl-CoA. Acetyl-CoA then enters the TCA cycle, where carbon intermediates and reducing equivalents are generated to support cellular metabolism [14]. Saline–alkali stress can markedly alter glycolysis and TCA-cycle metabolism, thereby disturbing central carbon metabolism [15]. Maintaining carbon metabolic homeostasis is therefore important for plant adaptation to saline–alkali environments [16].

Brassinosteroids (BRs) are important regulators of plant stress resistance and regulate diverse aspects of plant growth, development, and environmental adaptation [17]. Among them, 24-epibrassinolide (EBR) is a highly active BR with well-established biological activity [18]. Application of exogenous EBR has been reported to alleviate various abiotic stresses, including salinity, drought, and heavy-metal stress [19]. EBR improves photosynthetic performance and pigment stability [20]. It also enhances antioxidant defense, osmotic adjustment, and ion homeostasis [7], reduces Na^+^ accumulation and Na^+^/K^+^ ratios, and modulates signaling pathways [17]. In cucumber exposed to salt or NaHCO_3_ stress, EBR alleviates growth inhibition, improves morphology, and maintains photosynthetic efficiency [21]. It also promotes the accumulation of osmolytes, such as soluble sugars and proline [22], and helps maintain redox balance [7,23]. EBR has also been associated with adjustments in carbon metabolism in plants exposed to abiotic stress [17]. It can enhance photosynthetic carbon assimilation by improving Calvin-cycle efficiency and Rubisco activity [24] and influence carbohydrate and respiratory metabolism under abiotic stress [17,19,25]. However, despite these documented protective effects [7,21,22,23], the effects of EBR on carbohydrate metabolism in cucumber leaves and roots under NaHCO_3_ stress remain poorly understood. In particular, its effects on glycolysis, organic acid metabolism, and the TCA cycle require further investigation. The present study therefore extends previous work by focusing specifically on these carbon-metabolic responses in cucumber leaves and roots.

Considering the importance of carbon metabolism for plant adaptation under abiotic stress [9,16], we hypothesized that EBR alleviates NaHCO_3_-induced injury in cucumber seedlings in association with adjustments in photosynthetic performance and carbon metabolism in leaves and roots. To test this hypothesis, we investigated seedling growth and photosynthetic performance; the accumulation of starch, sucrose, glucose, and fructose in leaves and roots; the activities of carbohydrate-metabolism-related, glycolytic, and TCA-cycle-associated enzymes; organic acid contents; and the early transcriptional responses of genes associated with glycolysis and the TCA cycle under NaHCO_3_ stress with or without EBR application. By integrating these physiological, biochemical, and transcriptional responses, this study aimed to clarify how EBR is associated with the adjustment of carbon metabolism during cucumber responses to NaHCO_3_ stress.

## 2. Results

**Figure 1:**
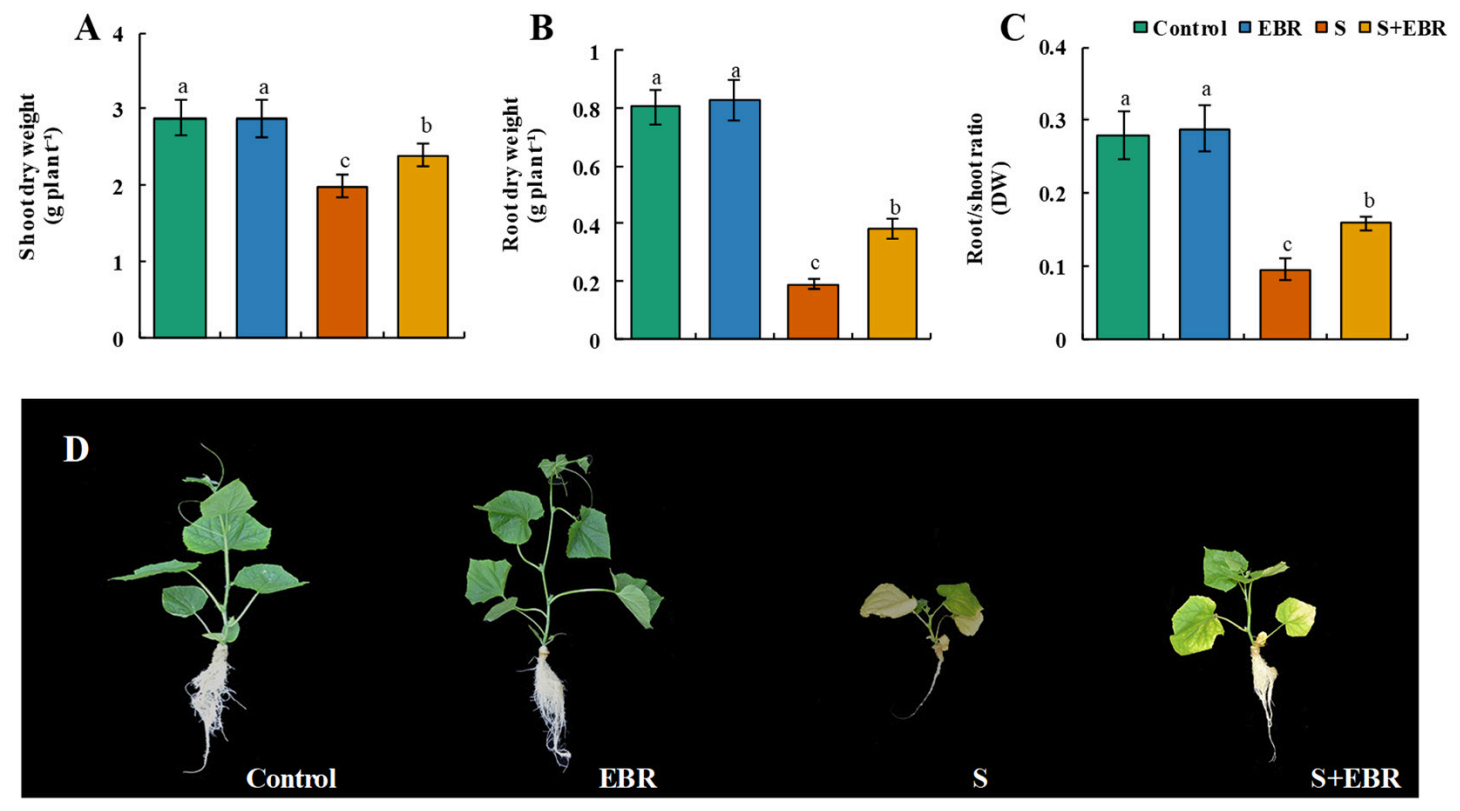
Effects of 24-epibrassinolide (EBR) on cucumber seedling growth and biomass accumulation in response to NaHCO_3_ stress. (**A**) Shoot dry weight. (**B**) Root dry weight. (**C**) Root-to-shoot dry weight ratio. (**D**) Representative phenotypes after 15 d of treatment. CK, plants supplied with Hoagland nutrient solution; EBR, plants supplied with Hoagland nutrient solution supplemented with 0.2 μM EBR; S, plants treated with 30 mM NaHCO_3_; S + EBR, plants treated with both 30 mM NaHCO_3_ and 0.2 μM EBR. Data are presented as means ± SD of three biological replicates (*n* = 3). Different lowercase letters indicate significant differences among treatments at *p* < 0.05.

NaHCO_3_ treatment markedly suppressed cucumber seedling growth, accompanied by pronounced leaf chlorosis and wilting (Figure 1D). In contrast, seedlings receiving EBR together with NaHCO_3_ exhibited less visible injury. NaHCO_3_ treatment significantly reduced shoot and root dry weights and the root-to-shoot dry weight ratio compared with the control, whereas EBR application significantly alleviated these reductions under NaHCO_3_ stress (*p* < 0.05; Figure 1A–C).

**Figure 2:**
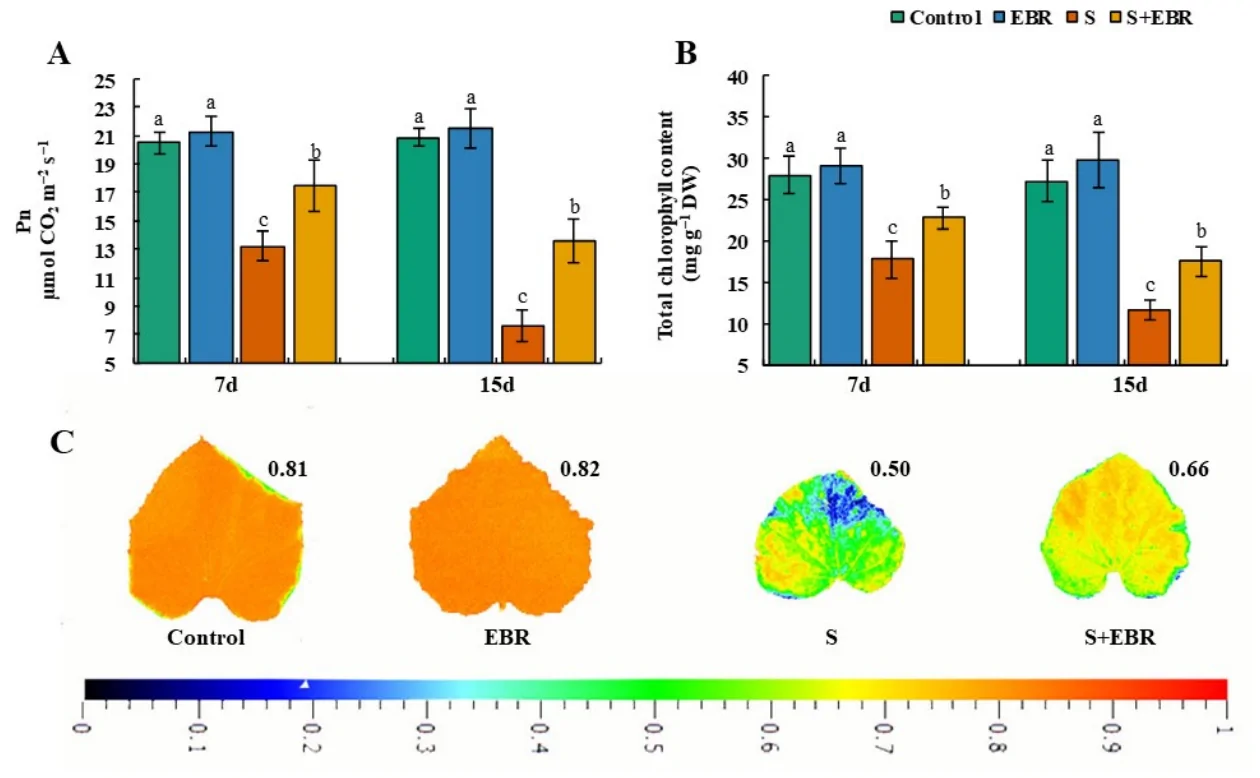
Effects of exogenous EBR on photosynthetic performance and chlorophyll status of cucumber seedlings exposed to NaHCO_3_ stress. (**A**) Net photosynthetic rate (Pn) measured at 7 and 15 d after treatment. (**B**) Total chlorophyll content determined at 7 and 15 d after treatment. (**C**) Representative false-color images of Fv/Fm obtained after 15 d of treatment. CK, Hoagland nutrient solution; EBR, Hoagland solution containing 0.2 μM EBR; S, 30 mM NaHCO_3_; S + EBR, combined treatment with 30 mM NaHCO_3_ and 0.2 μM EBR. Data in panels A and B are presented as means ± SD of three biological replicates (*n* = 3). Different lowercase letters indicate significant differences among treatments at *p* < 0.05. Panel (**C**) shows representative false-color Fv/Fm images.

Under control conditions, EBR had no significant effect on the net photosynthetic rate (Pn) or total chlorophyll content of cucumber seedlings. NaHCO_3_ treatment significantly reduced both parameters, whereas EBR application significantly alleviated these reductions (*p* < 0.05; Figure 2A,B). False-color fluorescence images also showed lower Fv/Fm under NaHCO_3_ stress. Seedlings treated with EBR showed relatively higher Fv/Fm under stress (Figure 2C).

**Figure 3:**
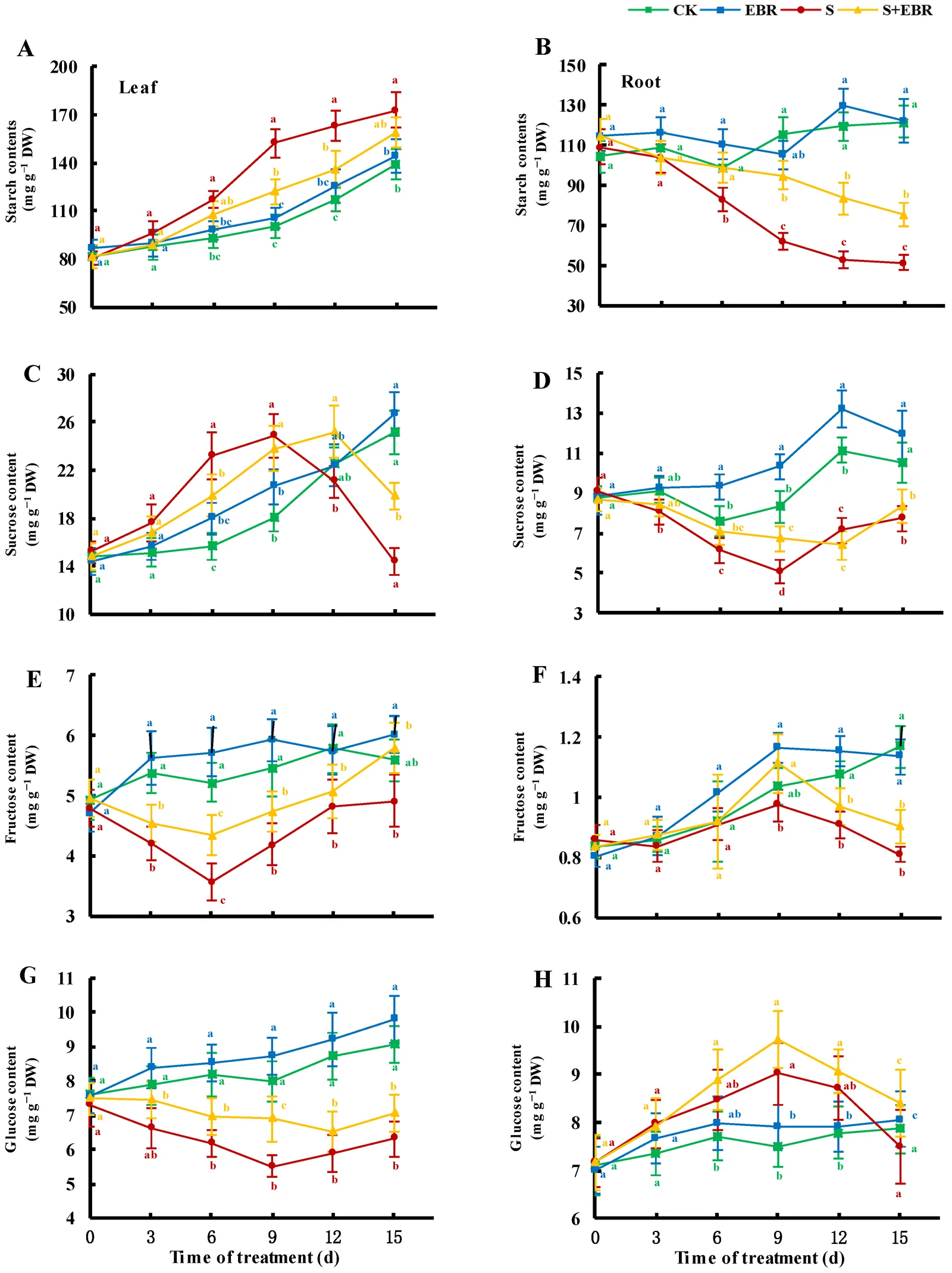
Effects of EBR on carbohydrate contents in leaves and roots of cucumber seedlings exposed to NaHCO_3_ stress. Starch contents in leaves (**A**) and roots (**B**), sucrose contents in leaves (**C**) and roots (**D**), fructose contents in leaves (**E**) and roots (**F**), and glucose contents in leaves (**G**) and roots (**H**). CK, Hoagland nutrient solution; EBR, Hoagland solution containing 0.2 μM EBR; S, 30 mM NaHCO_3_; S + EBR, 30 mM NaHCO_3_ combined with 0.2 μM EBR. Data are presented as means ± SD of three biological replicates (*n* = 3). Different lowercase letters indicate significant differences among treatments within the same sampling time at *p* < 0.05.

Under NaHCO_3_ stress, starch showed contrasting patterns between leaves and roots, progressively accumulating in leaves while declining in roots as the stress period extended. Sucrose content in leaves initially increased and subsequently decreased, whereas the opposite pattern was observed in roots. Leaf glucose and fructose contents initially declined and then partially recovered, while their levels in roots increased at the initial stage of stress before subsequently decreasing. EBR reduced excessive starch accumulation in leaves while increasing starch content in roots relative to NaHCO_3_ stress alone. EBR also markedly modified the stress-induced changes in sucrose, glucose, and fructose contents in both tissues. These results indicate that EBR mitigates NaHCO_3_-induced alterations in carbohydrate accumulation in leaves and roots (Figure 3).

**Figure 4:**
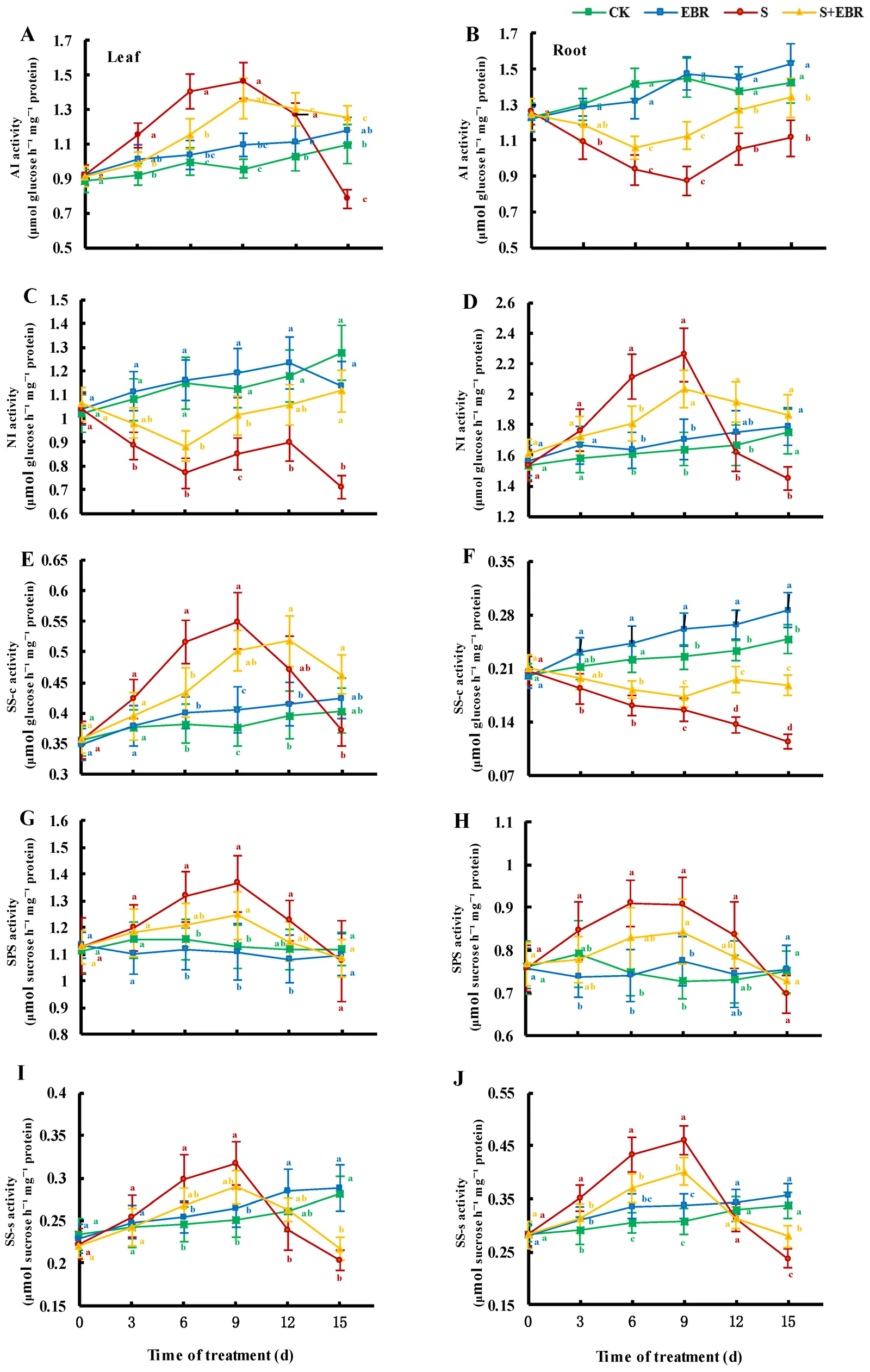
Effects of EBR on sucrose-metabolizing enzyme activities in leaves and roots of cucumber seedlings exposed to NaHCO_3_ stress. (**A**,**B**) Acid invertase (AI) activity. (**C**,**D**) Neutral invertase (NI) activity. (**E**,**F**) Sucrose synthase activity in the cleavage direction (SS-c). (**G**,**H**) Sucrose phosphate synthase (SPS) activity. (**I**,**J**) Sucrose synthase activity in the synthesis direction (SS-s). For each pair of panels, the left and right panels correspond to leaves and roots, respectively. CK, Hoagland nutrient solution; EBR, Hoagland nutrient solution containing 0.2 μM EBR; S, 30 mM NaHCO_3_; S + EBR, 30 mM NaHCO_3_ combined with 0.2 μM EBR. Data are presented as means ± SD of three biological replicates (*n* = 3). Different lowercase letters indicate significant differences among treatments within the same sampling time at *p* < 0.05.

NaHCO_3_ stress induced distinct temporal changes in sucrose-metabolizing enzyme activities in cucumber leaves and roots (Figure 4). In leaves, AI, SS-c, SPS, and SS-s activities increased at the early and middle stages of stress and subsequently declined, whereas NI activity generally decreased. In roots, AI and SS-c activities decreased during the early stage of stress, while NI, SPS, and SS-s activities initially increased and subsequently declined with prolonged stress.

EBR modified these stress-induced responses. In leaves, EBR reduced the NaHCO_3_-induced increases in AI, SS-c, SPS, and SS-s activities at the early and middle stages of treatment, while alleviating the decline in NI activity. At the later stage of stress, EBR maintained higher AI, SS-c, and SS-s activities than NaHCO_3_ stress alone. In roots, EBR alleviated the stress-induced decreases in AI and SS-c and modulated the responses of NI, SPS, and SS-s, particularly during prolonged stress. These results suggest that EBR modulates the activities of enzymes involved in sucrose cleavage and synthesis under NaHCO_3_ stress.

**Figure 5:**
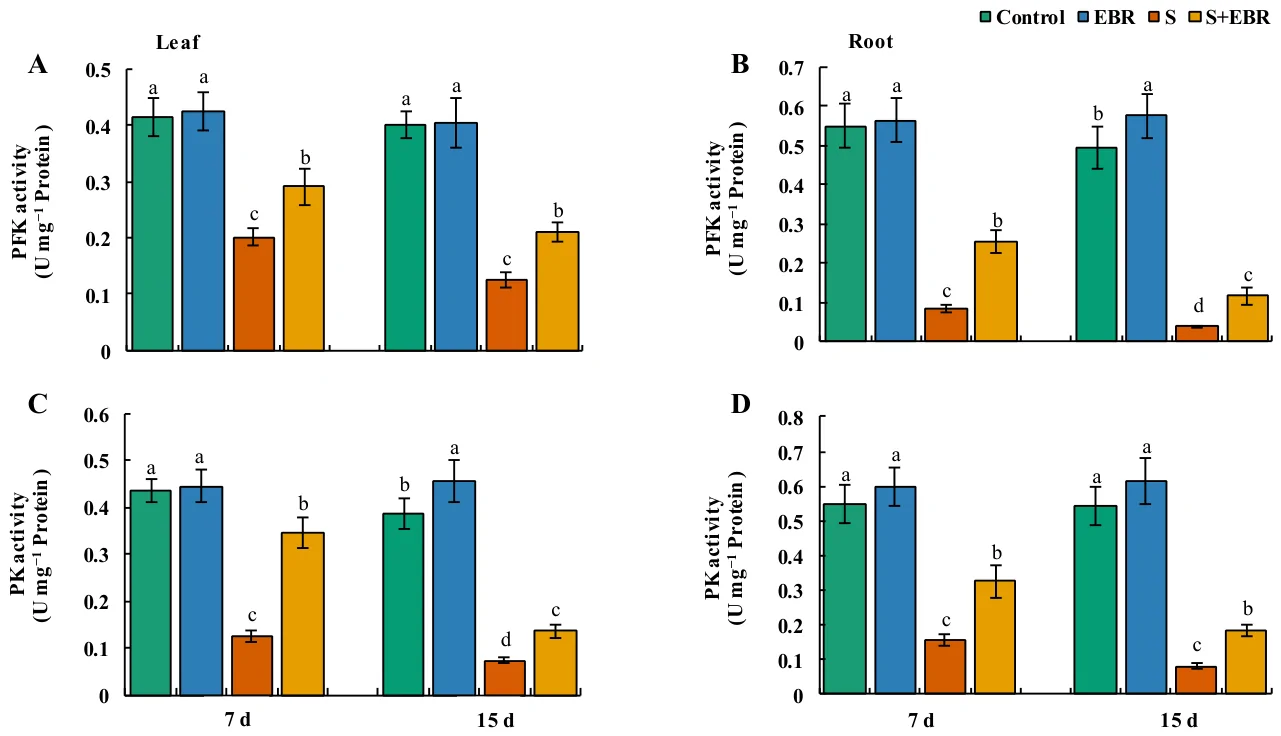
Effects of EBR on glycolytic enzyme activities in leaves and roots of cucumber seedlings exposed to NaHCO_3_ stress. (**A**,**B**) Phosphofructokinase (PFK) activities in leaves and roots, respectively. (**C**,**D**) Pyruvate kinase (PK) activities in leaves and roots, respectively. Measurements were performed after 7 and 15 d of different treatments. CK, Hoagland solution; EBR, Hoagland solution containing 0.2 μM EBR; S, 30 mM NaHCO_3_; S + EBR, 30 mM NaHCO_3_ combined with 0.2 μM EBR. Data are presented as means ± SD of three biological replicates (*n* = 3). Different lowercase letters indicate significant differences among treatments within the same sampling time at *p* < 0.05.

NaHCO_3_ treatment significantly reduced PFK and PK activities in both leaves and roots at 7 and 15 d compared with the control (*p* < 0.05; Figure 5). EBR application significantly alleviated these reductions under NaHCO_3_ stress at both sampling times (*p* < 0.05). Although PFK and PK activities in the S + EBR treatment remained below the control levels at 15 d, they were significantly higher than those under NaHCO_3_ stress alone (*p* < 0.05). These results indicate that EBR partially alleviated the NaHCO_3_-induced inhibition of key glycolytic enzyme activities.

**Figure 6:**
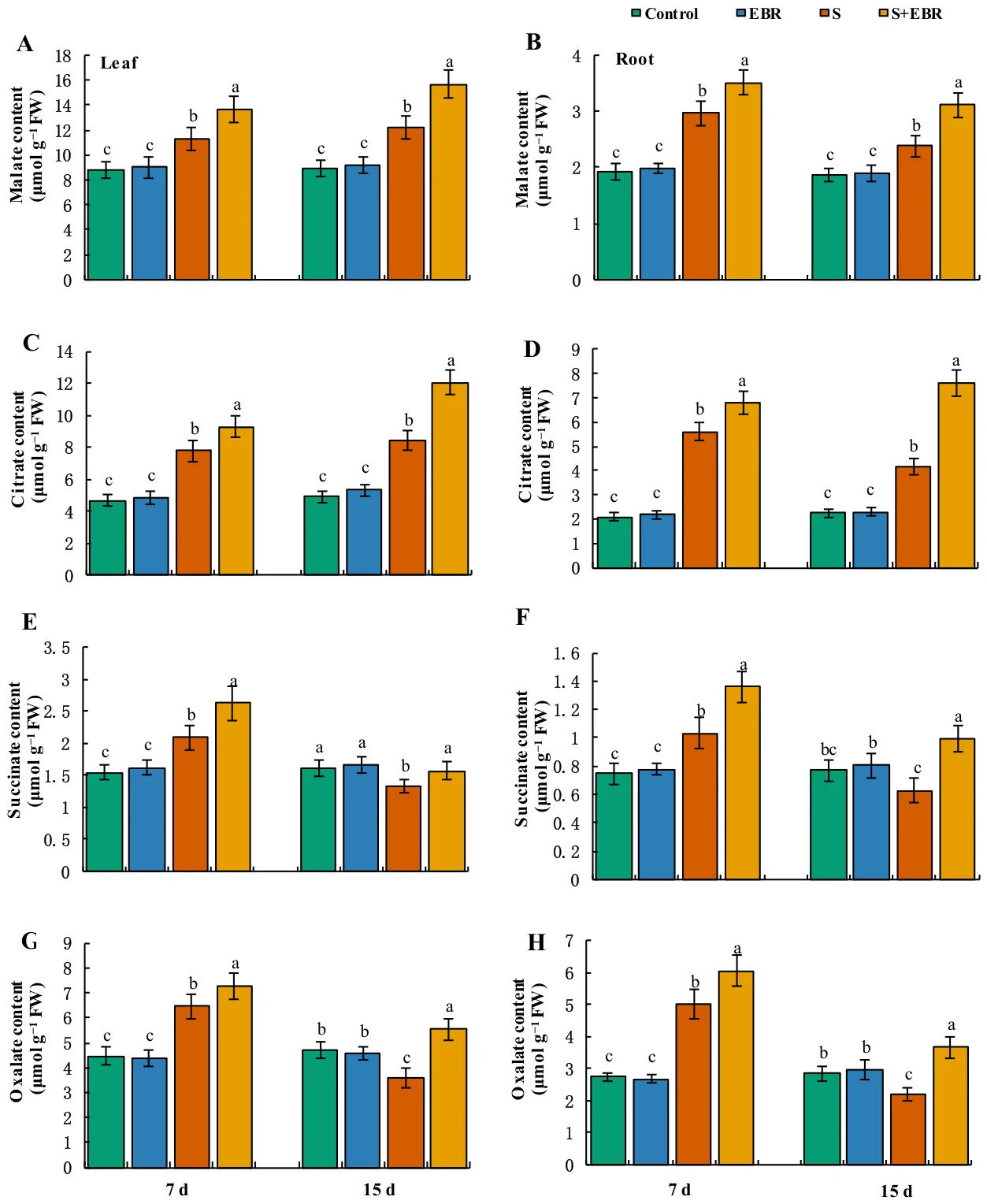
Effects of EBR on organic acid contents in leaves and roots of cucumber seedlings exposed to NaHCO_3_ stress. (**A**,**B**) Malic acid contents in leaves and roots, respectively. (**C**,**D**) Citric acid contents in leaves and roots, respectively. (**E**,**F**) Succinic acid contents in leaves and roots, respectively. (**G**,**H**) Oxalic acid contents in leaves and roots, respectively. CK, Hoagland solution; EBR, Hoagland solution containing 0.2 μM EBR; S, 30 mM NaHCO_3_; S + EBR, 30 mM NaHCO_3_ combined with 0.2 μM EBR. Data are presented as means ± SD of three biological replicates (*n* = 3). Different lowercase letters indicate significant differences among treatments within the same sampling time at *p* < 0.05.

After 7 d of NaHCO_3_ treatment, the contents of several measured organic acids increased in leaves and roots, although the responses varied among individual acids and tissues (Figure 6). At 15 d, the organic acids showed distinct compound- and tissue-specific responses. EBR also affected organic acid contents under NaHCO_3_ stress. However, the direction and statistical significance of these changes varied with the organic acid, tissue, and sampling time (*p* < 0.05).

**Figure 7:**
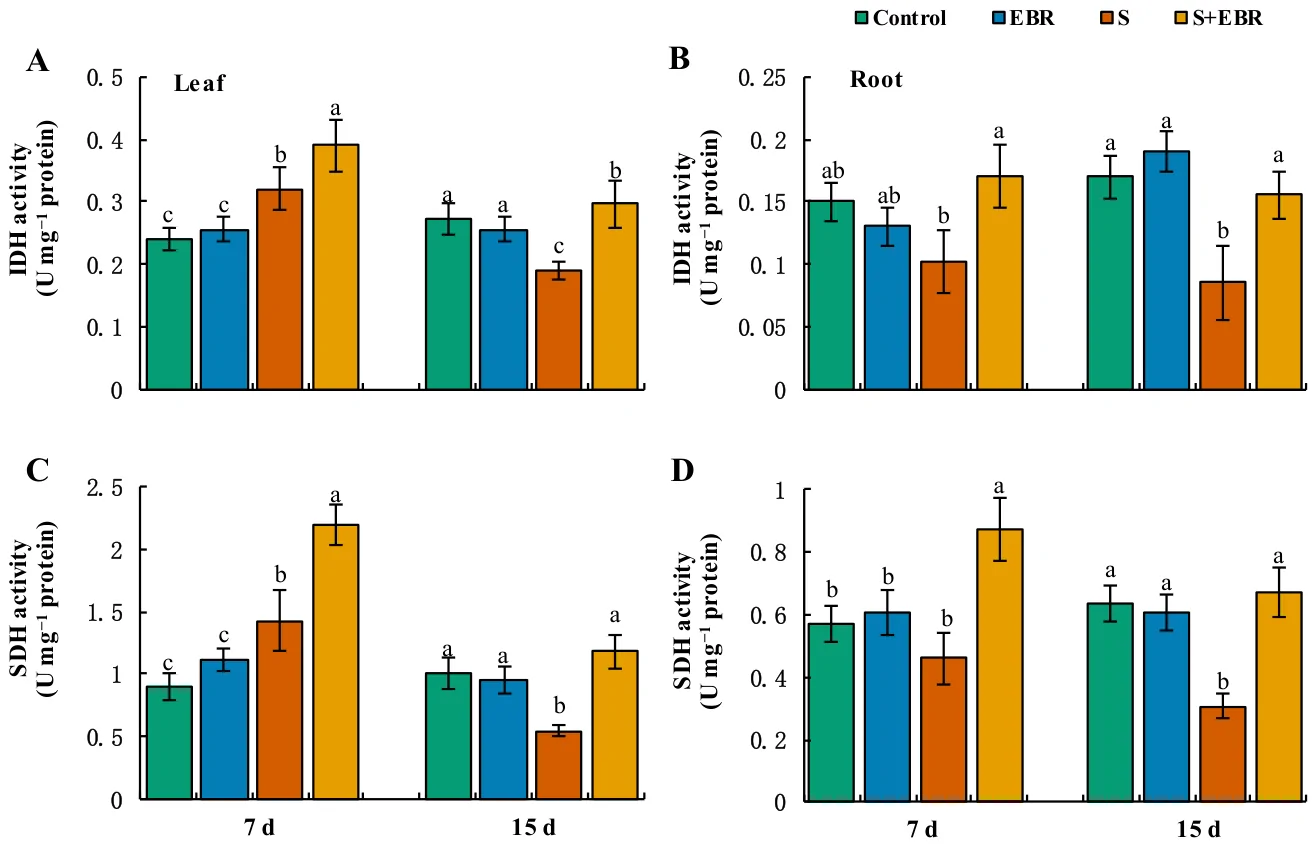
Effects of EBR on key enzyme activities associated with the tricarboxylic acid (TCA) cycle in leaves and roots of NaHCO_3_-stressed cucumber seedlings. (**A**,**B**) Isocitrate dehydrogenase (IDH) activities measured in leaves and roots, respectively. (**C**,**D**) Succinate dehydrogenase (SDH) activities measured in leaves and roots, respectively. CK, Hoagland solution; EBR, Hoagland solution containing 0.2 μM EBR; S, 30 mM NaHCO_3_; S + EBR, 30 mM NaHCO_3_ combined with 0.2 μM EBR. Data are presented as means ± SD of three biological replicates (*n* = 3). Different lowercase letters indicate significant differences among treatments within the same sampling time at *p* < 0.05.

At 7 d, NaHCO_3_ induced tissue-dependent changes in IDH and SDH activities, whereas prolonged NaHCO_3_ treatment significantly reduced the activities of both enzymes in leaves and roots at 15 d (*p* < 0.05; Figure 7). EBR application significantly increased IDH and SDH activities relative to NaHCO_3_ stress alone at both sampling times (*p* < 0.05).

**Figure 8:**
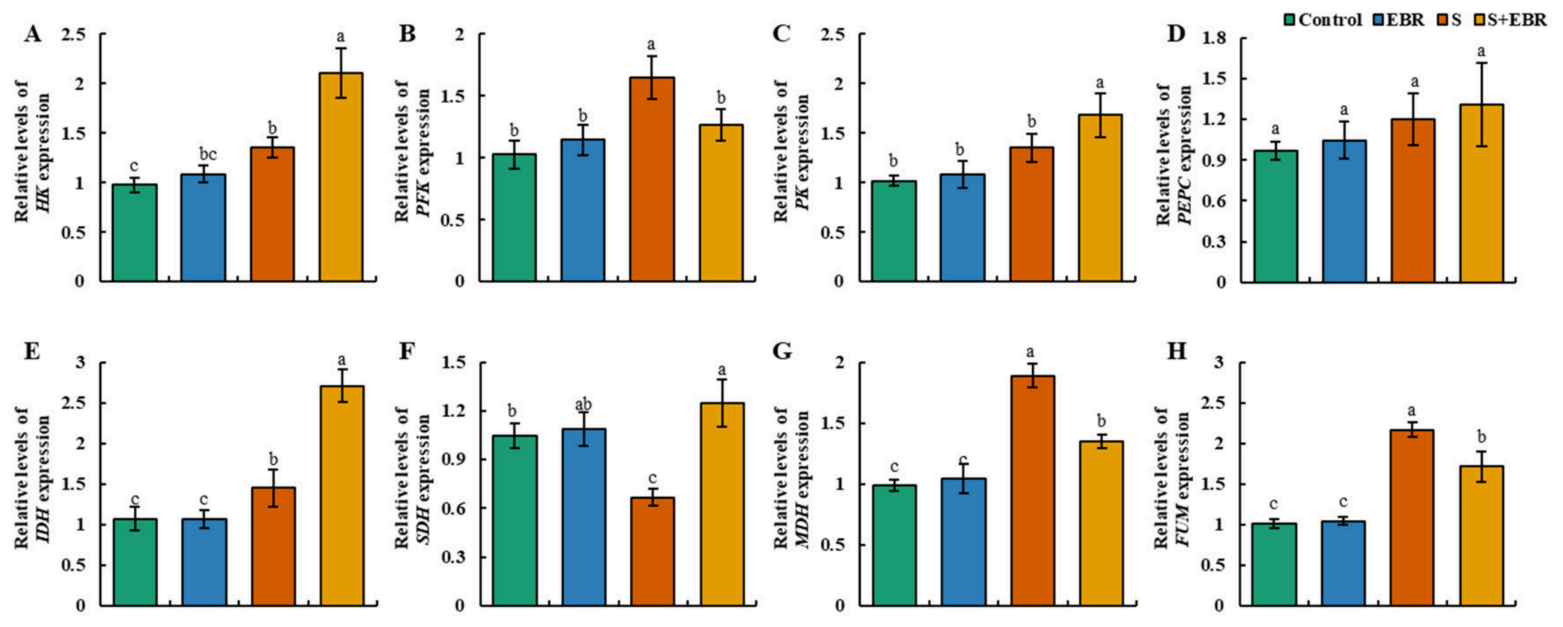
Effects of EBR on glycolysis- and TCA cycle-related gene expression in cucumber roots exposed to NaHCO_3_ stress. (**A**) *HK* (*hexokinase*). (**B**) *PFK* (*phosphofructokinase*). (**C**) *PK* (*pyruvate kinase*). (**D**) *PEPC* (*phosphoenolpyruvate carboxylase*). (**E**) *IDH* (*isocitrate dehydrogenase*). (**F**) *SDH* (*succinate dehydrogenase*). (**G**) *MDH* (*malate dehydrogenase*). (**H**) *FUM* (*fumarase*). Root samples were collected after 24 h of treatment. CK, Hoagland solution; EBR, Hoagland solution containing 0.2 μM EBR; S, 30 mM NaHCO_3_; S + EBR, 30 mM NaHCO_3_ combined with 0.2 μM EBR. Data are presented as means ± SD of three biological replicates *(n* = 3). Different lowercase letters indicate significant differences among treatments within the same sampling time at *p* < 0.05.

NaHCO_3_ stress significantly affected the expression of several glycolysis- and TCA cycle-associated genes in cucumber roots (*p* < 0.05). Relative to the control, *HK*, *PFK*, and *PK* transcript levels increased significantly, whereas PEPC expression did not differ significantly among treatments. Among the TCA cycle-associated genes, *MDH* and *FUM* were significantly upregulated and *SDH* was significantly downregulated by NaHCO_3_ treatment (*p* < 0.05), whereas *IDH* expression did not differ significantly from the control. EBR further altered these transcriptional responses under NaHCO_3_ stress, with gene-specific patterns. Collectively, these findings indicate that EBR alters the early transcriptional responses of glycolytic and TCA cycle-associated genes in cucumber roots under NaHCO_3_ stress (Figure 8).

## 3. Discussion

Carbohydrates constitute the major products of photosynthetic carbon fixation and can be broadly classified into two categories. Structural components, such as lignin and cellulose, mainly contribute to plant morphogenesis [26], whereas non-structural carbohydrates, including sucrose, fructose, glucose, and starch, primarily support metabolic processes. Saline–alkali stress suppresses photosynthesis, alters assimilate accumulation, and can disturb source–sink relationships [27,28]. Under salinity–alkalinity stress, carbohydrates function not only as osmotic regulators and structural components but also as signaling mediators involved in the regulation of gene expression and cellular metabolism [29,30]. The maintenance of carbohydrate metabolic homeostasis is closely associated with plant stress tolerance [11,31]. Therefore, investigating changes in carbon metabolism in cucumber seedlings under NaHCO_3_ stress is important for understanding their metabolic responses to alkaline conditions. Sucrose is a major transport sugar in many plant species [11,26,32]. In cucurbits, however, raffinose family oligosaccharides (RFOs), particularly raffinose and stachyose, also contribute substantially to long-distance carbohydrate transport [33]. In this study, leaf starch progressively accumulated under NaHCO_3_ stress. Leaf sucrose content initially increased and then declined, whereas fructose and glucose contents showed the opposite trend. In roots, starch content declined continuously, while sucrose first decreased and then increased. Root fructose and glucose increased at the early stage but declined thereafter. These results show that carbohydrate accumulation responded differently to NaHCO_3_ stress in leaves and roots. However, changes in assimilate transport between the two tissues cannot be confirmed because carbohydrate transport flux was not measured. In addition, RFOs were not determined in this study, and therefore the measured sugars do not represent the complete profile of transport carbohydrates in cucumber.

Exogenous EBR alleviated the inhibition of photosynthesis caused by NaHCO_3_ stress and altered carbohydrate accumulation in leaves and roots. EBR reduced starch accumulation in leaves and increased starch content in roots relative to NaHCO_3_ stress alone, and also modified the contents of sucrose, glucose, and fructose in both tissues. These results suggest that EBR partially alleviated NaHCO_3_-induced changes in carbohydrate accumulation, although its effects on carbohydrate transport between leaves and roots remain unclear. Similar effects of EBR on photosynthesis and carbon metabolism have been reported previously [18,19].

Changes in carbohydrate synthesis, transport, and accumulation are coordinated by multiple enzymes. Sucrose metabolism is primarily regulated by invertases (IV, AI, and NI), sucrose synthase (SS), and sucrose phosphate synthase (SPS) [10,11,33]. AI and NI catalyze sucrose hydrolysis into glucose and fructose in the vacuole and cytoplasm, respectively. Previous studies have shown that sucrose metabolism under abiotic stress is predominantly regulated through the SPS pathway [11,34]. Salinity–alkalinity stress alters the activities of carbon metabolism-related enzymes, thereby disrupting carbohydrate metabolism in cucumber [28,35]. In the present study, sucrose accumulation in leaves during the early stage of NaHCO_3_ stress was accompanied by increased SPS and AI activities. These changes suggest that sucrose metabolism was affected by NaHCO_3_ stress. In the roots, the decline in sucrose content was accompanied by changes in sucrose-metabolizing enzyme activities. During the early stage of stress, AI and SS-c activities decreased, whereas SS-s activity increased. However, SS-s activity declined as stress persisted. As stress persisted, leaf sucrose content declined, accompanied by decreases in AI, SS-c, and SS-s activities, while SPS activity gradually returned toward the control level. In contrast, the subsequent accumulation of sucrose in the roots was associated with increased AI activity and reduced SS-c, SS-s, and SPS activities. Exogenous EBR altered NI and SPS activities in cucumber leaves under NaHCO_3_ stress. These changes may be associated with the effects of EBR on sucrose metabolism. Moreover, EBR affected the activities of invertases, SS-c, SS-s, and SPS, which may contribute to changes in carbohydrate metabolism under NaHCO_3_ stress.

Glycolysis is a central metabolic pathway linking carbohydrate metabolism to the tricarboxylic acid (TCA) cycle and provides pyruvate and other intermediates for downstream metabolism [36,37,38]. Phosphofructokinase (PFK) and pyruvate kinase (PK) are the key rate-limiting and regulatory enzymes of glycolysis, respectively, and changes in their activities reflect the metabolic status of the glycolytic pathway [39,40].

In this study, NaHCO_3_ stress significantly reduced PFK and PK activities in cucumber leaves and roots. Previous studies have also reported changes in glycolytic enzyme activities under prolonged saline–alkali stress [28,39,41].

Meanwhile, the expression of glycolysis-related genes, including *HK*, *PFK*, and *PK*, was altered at 24 h. This suggests an early transcriptional response to NaHCO_3_ stress. The induction of some of these genes may reflect a compensatory response to metabolic perturbation [42,43,44,45]. However, these transcriptional changes cannot directly explain the changes in PFK and PK activities observed at 7 and 15 d because of the different sampling times. In addition, transcript abundance does not necessarily correspond to protein abundance or enzyme activity due to post-transcriptional and post-translational regulation [44,45]. Under prolonged stress, enzyme activities may also be affected by metabolic disturbance and oxidative damage [14]. EBR altered the early transcriptional responses of glycolysis-related genes and partially restored PFK and PK activities at later stages, suggesting its involvement in the adjustment of glycolytic metabolism under NaHCO_3_ stress. Further measurements of protein abundance and enzyme activity at the same sampling times are needed to clarify these relationships.

Reduced carbon assimilation and metabolic disturbance are major contributors to growth inhibition and biomass reduction under abiotic stress [33,36,46]. Carbohydrates provide carbon substrates required for plant growth, and reduced carbon assimilation under abiotic stress can contribute to biomass reduction [47]. Our results showed that exogenous EBR significantly mitigated the NaHCO_3_-induced inhibition of photosynthesis and growth, which was closely associated with its ability to stabilize carbon metabolism. However, carbon metabolism under saline–alkali stress is highly dynamic and complex, and further studies are required to elucidate the mechanisms by which EBR regulates carbohydrate metabolism in cucumbers under alkaline stress.

Glycolysis produces pyruvate, which can be converted to acetyl-CoA. Acetyl-CoA then enters the TCA cycle [14,38,48]. The TCA cycle is a central pathway of carbon metabolism and provides intermediates for multiple cellular processes [36]. Previous studies have shown that saline–alkali stress can alter TCA-cycle-associated metabolism in plants [28,39]. ROS accumulation has been reported to inhibit enzymes associated with the TCA cycle. In our previous study, EBR alleviated oxidative damage and enhanced antioxidant capacity in cucumber seedlings exposed to NaHCO_3_ stress [49]. Although oxidative stress was not directly assessed in the present study, these previous findings provide a possible physiological context for the partial recovery of IDH and SDH activities observed following EBR application.

Organic acids can contribute to cellular pH regulation and ionic homeostasis under saline–alkali stress [50]. In the present study, NaHCO_3_ induced distinct temporal changes in individual organic acids in cucumber leaves and roots. These changes suggest that organic acid metabolism responds dynamically to NaHCO_3_ stress. Similar stress-associated changes in organic acid metabolism have also been reported in other plant species [51,52,53]. These changes may contribute to physiological adaptation to the high-pH and ionic components of NaHCO_3_ stress; however, ionic composition was not directly measured in the present study. In our previous work, EBR improved ion homeostasis in cucumber seedlings exposed to NaHCO_3_ stress, including the regulation of Na^+^ and K^+^ accumulation [6]. These previous findings provide relevant physiological context for the organic-acid responses observed here, although a direct causal relationship between organic acid accumulation and ion homeostasis cannot be established from the present data.

Exogenous EBR altered organic acid accumulation under NaHCO_3_ stress, with responses varying among individual organic acids, tissues, and sampling times. Two key enzymes of the plant TCA cycle are isocitrate dehydrogenase (IDH) and succinate dehydrogenase (SDH) [51,54]. Moreover, TCA cycle-related genes showed distinct transcriptional responses at 24 h, indicating an early adjustment of TCA-associated metabolism under NaHCO_3_ stress. EBR altered the expression patterns of these genes during this early response. Because gene expression and enzyme activities were measured at different time points, these transcriptional and biochemical responses cannot be directly linked. Nevertheless, early transcriptional changes and the partial recovery of IDH and SDH activities at later stages suggest that EBR may affect TCA-associated metabolism during NaHCO_3_ stress. Changes in organic acid metabolism may contribute to physiological adjustment under saline–alkali stress through their roles in cellular pH regulation and ionic homeostasis [55]. However, the physiological roles of these EBR-associated changes require further investigation. Additionally, organic acids function as osmotic regulators that help maintain water balance in plants exposed to saline–alkali stress.

Exogenous EBR partially alleviated the NaHCO_3_-induced changes in IDH and SDH activities and altered organic acid accumulation in cucumber leaves and roots. These responses indicate that EBR affected TCA-cycle-associated metabolism under NaHCO_3_ stress.

This study was conducted with one cucumber cultivar and single concentrations of EBR and NaHCO_3_ under hydroponic conditions. Because NaHCO_3_ simultaneously affects Na^+^, HCO_3_^−^, and solution pH, their individual effects could not be distinguished. In addition, carbohydrate transport and raffinose-family oligosaccharides were not measured, and gene expression and enzyme activities were determined at different sampling times. Further studies using different genotypes and treatment levels are needed to verify these responses under broader growth conditions.

**Figure 9:**
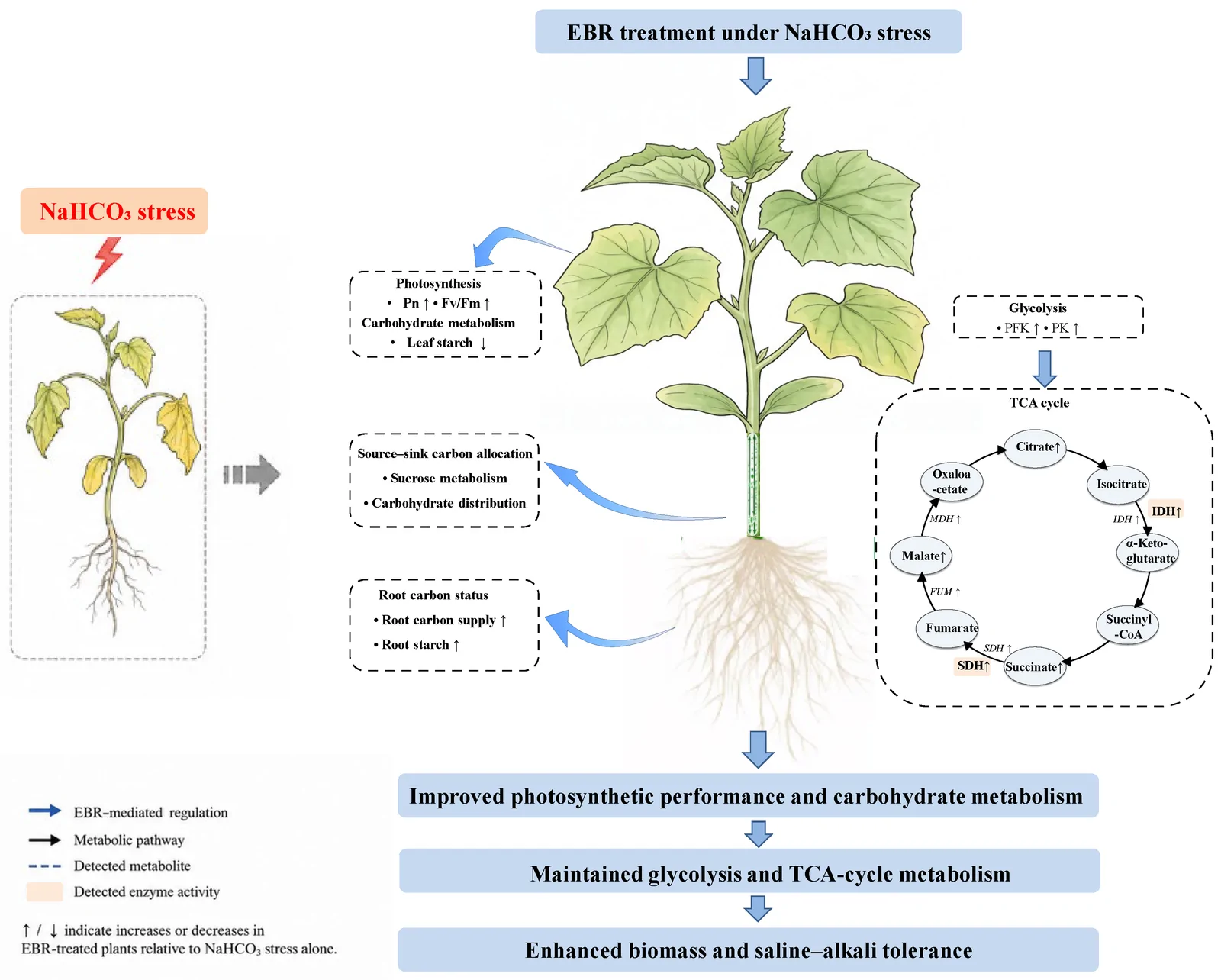
Proposed model of EBR-mediated changes in carbon metabolism in cucumber seedlings under NaHCO_3_ stress. NaHCO_3_ stress reduced photosynthetic performance and altered carbohydrate metabolism, glycolysis, TCA-cycle-associated metabolism, and organic acid accumulation. EBR alleviated these changes and improved seedling growth under NaHCO_3_ stress. Blue arrows indicate EBR-associated responses, and black arrows indicate metabolic pathways. Upward and downward arrows indicate increases and decreases, respectively, in the S + EBR treatment compared with the S treatment.

Figure 9 summarizes the observed effects of EBR on carbon metabolism under NaHCO_3_ stress. NaHCO_3_ stress reduced photosynthetic performance and altered carbohydrate contents, glycolytic enzyme activities, organic acid contents, and TCA-cycle-associated responses. EBR alleviated several of these changes. In particular, EBR reduced starch accumulation in leaves, increased starch content in roots, and altered soluble sugar contents in both tissues. EBR also partially restored the activities of key glycolytic and TCA-cycle-associated enzymes and altered the early expression of related genes.

NaHCO_3_ stress inhibited cucumber seedling growth and photosynthetic performance and altered carbohydrate metabolism. Exogenous EBR alleviated these effects and altered starch, sucrose, glucose, and fructose contents in leaves and roots. EBR also alleviated the inhibition of PFK, PK, IDH, and SDH activities. In addition, it altered organic acid contents and the early expression of glycolysis- and TCA-cycle-associated genes. These results suggest that EBR improves cucumber tolerance to NaHCO_3_ stress partly through changes in carbon metabolism. Because carbohydrate transport between leaves and roots was not directly measured, the tissue-specific changes observed here should not be interpreted as direct evidence of altered assimilate translocation.

## 4. Materials and Methods

### 4.1. Plant Materials and Treatments

Seeds of cucumber (*Cucumis sativus* L. cv. Jinyan No. 4) were soaked in water at 28 °C for 4 h and then disinfected with 0.3% sodium hypochlorite for 30 s. After thorough rinsing with distilled water, the seeds were germinated in darkness at 28 °C. Following radicle emergence, the germinated seeds were transferred into 50-cell trays filled with growth substrate and grown in a naturally illuminated greenhouse. When the cotyledons were fully expanded, uniform seedlings were carefully removed from the trays, and the roots were gently washed to remove the adhering substrate. The seedlings were individually secured with sponge supports in the holes of floating foam boards and transferred to the hydroponic system. They were initially maintained in water for 1 d, followed by half-strength Hoagland nutrient solution for 1 d and full-strength Hoagland nutrient solution for 6 d before treatment initiation. Four treatment groups were used: (1) CK, full-strength Hoagland nutrient solution alone; (2) EBR, 0.2 μM EBR added to the Hoagland nutrient solution; (3) S, 30 mM NaHCO_3_ added to the Hoagland nutrient solution; and (4) S + EBR, Hoagland nutrient solution supplemented with both 30 mM NaHCO_3_ and 0.2 μM EBR. EBR was purchased from Sigma-Aldrich (Burlington, MA, USA). A stock solution was prepared by dissolving 25 mg EBR in 10 mL absolute ethanol and then diluted with the nutrient solution to a final EBR concentration of 0.2 μM. The final ethanol concentration in the treatment solution was approximately 0.004% (*v*/*v*), and the same amount of ethanol was added to the EBR-free treatments. The EBR concentration (0.2 μM) was selected based on our previous studies in cucumber seedlings under NaHCO_3_ stress [6]. The initial pH and EC of the freshly prepared treatment solutions were measured before treatment. The pH values were approximately 6.1 for CK and EBR and 8.5 for S and S + EBR, while the corresponding EC values were approximately 2.3 and 4.6 mS cm^−1^, respectively. Plants were arranged according to a randomized block design, with 50 hydroponic containers assigned to each treatment. Each hydroponic container contained 6 L of Hoagland nutrient solution and was fitted with a floating foam board containing five planting holes, with one cucumber seedling secured in each hole using a sponge support. Each container was maintained as an independent hydroponic unit and received the nutrient solution corresponding to its designated treatment. For biochemical and gene-expression analyses, tissues collected from 10 individual plants were pooled to constitute one biological sample, and three independent biological replicates were analyzed for each treatment (*n* = 3).

During the experiment, plants were grown in a solar greenhouse under natural light and temperature conditions. The nutrient solutions were continuously aerated and renewed every 2 d. Depending on the measurements, the second and third fully expanded leaves below the shoot apex and/or roots were sampled at 0, 3, 6, 9, 12, and 15 d, or at 7 and 15 d after treatment. Leaves and roots at the same position were sampled from all treatments, and different plants were used at each sampling time. Young root tips were collected at 24 h for gene-expression analysis. All samples were immediately frozen in liquid nitrogen and stored at −80 °C until analysis. Plant phenotypes and chlorophyll fluorescence images were recorded at 15 d.

### 4.2. Determination of Plant Biomass

Plant biomass was determined 15 d after treatment. Five plants from each treatment were collected and thoroughly rinsed with deionized water. Shoots and roots were separated and initially heated at 105 °C for 15 min, followed by drying at 75 °C until a constant mass was obtained. Shoot and root dry weights were determined separately, and the root-to-shoot dry weight ratio was subsequently calculated.

### 4.3. Determination of Photosynthetic Parameters, Chlorophyll Content, and Chlorophyll Fluorescence

Photosynthetic measurements were conducted at 7 and 15 d after treatment using the second and third fully expanded leaves below the shoot apex. Net photosynthetic rate (Pn) was measured using a LI-6400 portable photosynthesis system (LI-COR, Lincoln, NE, USA) under natural illumination and ambient CO_2_ conditions. Three independent biological replicates were analyzed for each treatment.

Chlorophyll was extracted from fresh leaf tissue using 80% (*v*/*v*) acetone. The absorbance of the extracts was measured spectrophotometrically at 645 and 663 nm using 80% acetone as the blank. Chlorophyll a and chlorophyll b contents were calculated according to the method of Arnon (1949) [56], and total chlorophyll content was calculated as the sum of chlorophyll a and chlorophyll b.

Chlorophyll fluorescence was measured at 15 d after treatment using an Imaging-PAM chlorophyll fluorescence system (Heinz Walz GmbH, Effeltrich, Germany). Leaves were dark-adapted for 30 min before measurement, after which the maximum quantum efficiency of photosystem II (Fv/Fm) was determined. Representative false-color Fv/Fm images were recorded simultaneously.

### 4.4. Determination of Sucrose-Metabolizing Enzyme Activities

Sucrose-metabolizing enzyme activities were determined according to Lowell et al. (1989) [57], Hubbard et al. (1989) [58], and Hu et al. (2009) [59], with minor modifications. Fresh leaf or root tissue (1.0 g) was homogenized with four volumes of ice-cold 50 mM HEPES-NaOH buffer (pH 7.5) containing 10 mM MgCl_2_, 1 mM EDTA, 2.5 mM DTT, 0.1% (*v*/*v*) bovine serum albumin (BSA), and 0.05% (*v*/*v*) Triton X-100. After centrifugation at 13,000× *g* for 20 min, the supernatant was collected and dialyzed against HEPES–NaOH buffer before enzyme assays. Acid invertase (AI) and neutral invertase (NI) activities were determined from sucrose hydrolysis under acidic and neutral assay conditions, respectively. Sucrose synthase activity in the cleavage direction (SS-c) was determined from sucrose cleavage, whereas sucrose synthase in the synthesis direction (SS-s) and sucrose-phosphate synthase (SPS) activities were determined from sucrose formation using the corresponding reaction systems described in the cited methods [57,58,59]. Enzyme activities were normalized to soluble protein content.

### 4.5. Determination of Carbohydrate Contents

Carbohydrate contents were determined according to Yemm and Willis (1954) [60] and McCready et al. (1950) [61], with minor modifications. Leaf and root samples were heated at 105 °C for 15 min and subsequently dried at 70 °C to constant weight before being ground into a fine powder. Approximately 0.05 g of dried material was extracted with 80% (*v*/*v*) ethanol at 80 °C in a water bath. After centrifugation, the supernatant was collected for the determination of soluble carbohydrates. Sucrose, glucose, and fructose contents were determined colorimetrically using their corresponding standard curves. For sucrose determination, the contribution of free sugars in the extract was accounted for using the corresponding sample blank during color development. The ethanol-insoluble residue remaining after extraction of soluble sugars was used for starch determination. The residue was hydrolyzed under acidic conditions, and starch content was subsequently determined using the anthrone colorimetric method with a glucose standard curve. Carbohydrate contents were expressed on a dry-weight basis.

### 4.6. Determination of Organic Acid Contents

Organic acids were quantified by high-performance liquid chromatography (HPLC) based on the method of Han et al. (2019) [62], with minor modifications. Fresh leaf and root samples were homogenized separately in ultrapure water, followed by centrifugation. The collected supernatants were passed through 0.22 μm membrane filters before chromatographic analysis. Separation was achieved on a SunFire C18 column (250 mm × 4.6 mm, 5 μm; Waters, Milford, MA, USA) using an LC-20AT HPLC system (Shimadzu, Kyoto, Japan) equipped with a multi-wavelength UV–Vis detector. The mobile phase consisted of 0.01 M phosphate buffer containing 3.0% methanol (pH 2.8), delivered at a flow rate of 0.8 mL min^−1^. Chromatographic separation was performed at ambient temperature. A 10 μL aliquot of each sample was injected, and UV absorbance was monitored at 210 nm. Citric, malic, oxalic, and succinic acids were identified by matching their retention times with those of reference standards and quantified using external calibration curves.

### 4.7. Determination of Key Glycolytic Enzyme Activities

The activities of phosphofructokinase (PFK) and pyruvate kinase (PK) were determined according to Baud et al. (2007) [63] and Le Roux et al. (2006) [64], with minor modifications. Fresh leaf or root tissue (0.50 g) was homogenized in pre-chilled extraction buffer at 4 °C and centrifuged, and the resulting supernatant was used for enzyme assays. PFK activity was determined using a coupled enzymatic assay with fructose-6-phosphate and ATP as substrates, and the oxidation of NADH was monitored spectrophotometrically at 340 nm. PK activity was determined using a lactate dehydrogenase-coupled assay with phosphoenolpyruvate and ADP as substrates, with NADH oxidation monitored at 340 nm. Enzyme activities were calculated according to the corresponding assay procedures and normalized to soluble protein content.

### 4.8. Determination of Key TCA Cycle Enzyme Activities

The activities of NADP^+^-dependent isocitrate dehydrogenase (NADP-IDH) and succinate dehydrogenase (SDH) were determined using commercial assay kits (BC0400 and BC0950, respectively; Solarbio, Beijing, China) according to the manufacturer’s instructions. Fresh leaf and root tissues were homogenized under ice-cold conditions using the extraction buffers provided with the corresponding kits. After centrifugation, the supernatants were collected for enzyme activity assays. NADP-IDH activity was measured by monitoring NADPH formation at 340 nm using a UV–Vis spectrophotometer. SDH activity was determined by monitoring the reduction of 2,6-dichlorophenolindophenol (DCPIP) at 600 nm, with phenazine methosulfate (PMS) serving as an intermediate electron carrier. Enzyme activities were calculated according to the manufacturer’s instructions, with appropriate blank corrections.

### 4.9. Gene Expression Analysis

Young root tips were collected after 24 h of treatment. Total RNA was extracted from each biological sample using an RNA extraction kit according to the manufacturer’s instructions. RNA quality and concentration were checked before reverse transcription, and cDNA was synthesized using a reverse-transcription kit (Vazyme Biotech Co., Ltd., Nanjing, China). Quantitative real-time PCR (qRT-PCR) was performed on an ABI 7500 Real-Time PCR System (Applied Biosystems, Foster City, CA, USA) using ChamQ SYBR qPCR Master Mix (Vazyme Biotech Co., Ltd., Nanjing, China) and gene-specific primers (Table 1). The amplification program consisted of 94 °C for 30 s, followed by 40 cycles of 94 °C for 5 s and 60 °C for 34 s. Actin was used as the internal reference gene. Three independent biological replicates were analyzed for each treatment, with three technical replicates for each biological sample. Relative transcript abundance was calculated using the 2^−ΔΔCt t^ method [9].

### 4.10. Statistical Analysis

All data were analyzed using SPSS 19.0 (IBM, Armonk, NY, USA). Data are presented as the mean ± standard deviation (SD) of three biological replicates (*n* = 3). For comparisons among treatments at each sampling time, data were subjected to one-way analysis of variance (ANOVA), followed by Duncan’s multiple range test. Differences were considered statistically significant at *p* < 0.05. Microsoft Excel 2010 and GraphPad Prism 8.0 were used for data processing and figure preparation.

## Figures and Tables

**Table 1:** Primer sequences used for quantitative real-time PCR (qRT-PCR).

Gene Name	Forward Primer (5′–3′)	Reverse Primer (5′–3′)
*CsHK*	CATCTGCTTCATCAGGCGTGTTG	ATACCCGCAAACCTAGTCCAATCC
*CsPFK*	TGGTTACCGGCGGCGATAATC	TCAACAGTGGAATAGGCAGGATGG
*CsPK*	TGTGCTGAGTCTGAGAAGGTGTTC	TTCCAAGTGCGTCATAGGTTCCC
*CsPEPC*	ACTTCGTCTCCGTGATTCATACAT	GGTTTGCTTGCTTCCATTATTTCC
*CsIDH*	GGTCGCCAATCCCATCGTTGAG	CTGTGGCATCACGGTGTGGAAG
*CsMDH*	AGTCACCACTAGCAGCGGAGAG	GCAGCAGACAGAGCACTAGATAGC
*CsSDH*	AATAATGAATAGTGATGGTAGC	CAAGTATGAGCAGAGGTT
*CsFUM*	TGCCACACCTCTAACTCTTG	CAACAGCCGTTCCACCTT
*Actin*	ATGGCCGATGCCGAGGATAT	TAGGAGCATCATCACCAGCAAAAC

## Data Availability

The data presented in this study are available from the corresponding author upon reasonable request.
